# High Curie temperature and enhanced magnetoelectric properties of the laminated Li_0.058_(Na_0.535_K_0.48_)_0.942_NbO_3_/Co_0.6_ Zn_0.4_Fe_1.7_Mn_0.3_O_4_ composites

**DOI:** 10.1038/srep44855

**Published:** 2017-03-24

**Authors:** Haibo Yang, Jintao Zhang, Ying Lin, Tong Wang

**Affiliations:** 1 School of Materials Science and Engineering, Shaanxi University of Science and Technology, Weiyang, Xi’an, Shaanxi 710021, PR China

## Abstract

Laminated magnetoelectric composites of Li_0.058_(Na_0.535_K_0.48_)_0.942_NbO_3_ (LKNN)/Co_0.6_Zn_0.4_Fe_1.7_Mn_0.3_O_4_ (CZFM) prepared by the conventional solid-state sintering method were investigated for their dielectric, magnetic, and magnetoelectric properties. The microstructure of the laminated composites indicates that the LKNN phase and CZFM phase can coexist in the composites. Compared with the particulate magnetoelectric composites, the laminated composites have better piezoelectric and magnetoelectric properties due to their higher resistances and lower leakage currents. The magnetoelectric behaviors lie on the relative mass ratio of LKNN phase and CZFM phase. The laminated composites possess a high Curie temperature (*T*_*C*_) of 463 °C, and the largest ME coefficient of 285 mV/cm Oe, which is the highest value for the lead-free bulk ceramic magnetoelectric composites so far.

Magnetoelectric (ME) materials have drawn a continuously increasing interest because of their potential applications as multifunctional devices^1^,^2^,^3^,^4^,^5^,^6^,^7^,^8^. These materials simultaneously exhibit ferroelectricity and ferromagnetism. In multiferroic materials, the coupling interaction between different order parameters could produce new effects, such as ME effect. The ME effect is characterized as an induced electric polarization under an applied magnetic field or an induced magnetization under an external electric field in the multiferroics, which can be expressed by a parameter α_E_ = δE/δH^9^,^10^. There are typically two types in ME effects, direct and converse. Magnetic-field controlled polarization refers to the direct ME effect. Commonly, the ME effect in single phases is weak and appears at low temperature, leading to a difficulty in practical applications^11^. So the alternative artificial multiferroic composites are being extensively studied, which can be obtained by combining a ferroelectric phase and a ferromagnetic phase at room temperature, and these materials display properties of the parent compounds and their coupling. Neither the ferroelectric phase nor the magnetic phase has the ME effect, but the composites of these two phases have the remarkable ME effect. The ME effect in the composites is extrinsic, depending on the microstructure of the composites and the coupling interaction across ferroelectric-magnetic interfaces. These multiferroic composites have been found to exhibit a larger ME effect than that of the single-phase materials by more than one order of magnitude^12^.

The bulk ceramic ME composites could have various connectivity schemes, but the common connectivity schemes are as follow. (1) 0-3 type particulate ceramic composites where the ferroelectric/magnetic particles are embedded in a matrix of magnetic/ferroelectric phase, so the two phases are easy to produce some unpredictable phases during high temperature sintering and thus degrade the performance of the composites. Moreover, the large leakage current of the particulate composites makes the electric poling difficult and reduces the ME effect due to the low electric resistivities of the magnetic phases. (2) 2-2 type laminated ceramic composites consisting of the piezoelectric and magnetic oxide layers are scarce. The laminated ceramic composites have larger resistivity and lower leakage current due to the magnetic phase can be completely blocked by the high resistive ferroelectric layer, which is beneficial to the improvement of the ME effect. The laminated ceramic composites have much higher ME coefficient than those of single-phase materials and particulate composites^13^. It is known that the laminated composites made by gluing are very brittle^14^,^15^. Whereas, the laminated ceramic composites made by co-fire technique are solid and could be potentially available for low-temperature co-fired ceramics (LTCC) technology, which is beneficial to the miniature of magnetoelectric components and devices.

Currently, the bulk ceramic ME composite are mainly prepared by the composition of high piezoelectric lead zirconium titanate (PZT) and ferrite due to their excellent ME effect^16^,^17^. Recently, with the demand of environmental protection and the sustainable development of human society, developing new environmental friendly lead-free ME materials has become one of the hot investigation spots in the developed countries. At present, lead-free bulk ceramic ME composites are mainly based on BaTiO_3_-based piezoelectric materials. However, the sintering temperature of BaTiO_3_-based ferroelectric materials is approximately 1350 °C, which is much higher than that of spinel ferrite materials. It is hard to gain well dense BaTiO_3_-based bulk ceramic ME composites. Furthermore, high sintering temperature easily leads to the ion diffusion between the two phases. The diffusion of Fe ions from ferrite to ferroelectric phase would decrease the Curie temperature and even make tetragonal ferroelectric BaTiO_3_ phase transform into paraelectric hexagonal BaTiO_3_ phase^18^,^19^. The low Curie temperature of BaTiO_3_ leads to poor temperature stability of electrical properties. However, potassium sodium niobate (KNN)-based ceramics are considered as one of the promising lead-free candidates for its biocompatibility, high Curie temperature, excellent piezoelectric properties, making up for the deficiency of the low Curie point of BaTiO_3_ and providing excellent promise for widespread use^20^,^21^.

Recently, the (K_0.45_Na_0.55_)_0.98_Li_0.02_(Nb_0.77_Ta_0.18_Sb_0.05_)O_3_/Ni_0.37_Cu_0.20_Zn_0.43_Fe_1.92_O_3.88_ laminated bulk ceramic ME composites with a high ME coefficient of 133 mV/cm Oe and a Curie temperature of 240 °C has been reported by our group^22^. It can be found that the ME coefficient of (K_0.45_Na_0.55_)_0.98_Li_0.02_(Nb_0.77_Ta_0.18_Sb_0.05_)O_3_/Ni_0.37_Cu_0.20_Zn_0.43_Fe_1.92_O_3.88_ laminated bulk ceramic composites is not desirable due to the low magnetostrictive coefficient of NCZF phase. Moreover, although Ta and Sb can provide further improvement on the dielectric constant and piezoelectric coefficient, their use is not favored because of the high cost of Ta and the toxicity of Sb^23^. Hence it is necessary to develop a new environment-friendly KNN-based laminated ME composite with an improved ME coefficient. Simple Li-doped KNN is still a good candidate for use in the lead-free piezoelectric ceramics^24^,^25^. Compared with the classical cobalt ferrite (CFO), Zn and Mn doped cobalt ferrite (Co_0.6_Zn_0.4_Fe_1.7_Mn_0.3_O_4_) possess enhanced ferromagnetic and magnetostrictive properties^26^,^27^. Hence, Li_0.058_(Na_0.535_K_0.48_)_0.942_NbO_3_ (LKNN) was chosen to be the ferroelectric phase and Co_0.6_Zn_0.4_Fe_1.7_Mn_0.3_O_4_ (CZFM) with 0.3 wt% Li_2_CO_3_ was used as the magnetic phase. They both have a sintering temperature of 1030 °C. The ME coefficient of the as-prepared LKNN/CZFM composites is as high as 285 mV/cm Oe. To the best of our knowledge, this value is the highest for the lead-free bulk ceramic ME composites so far.

## Results and Discussion

**Figure 1:**
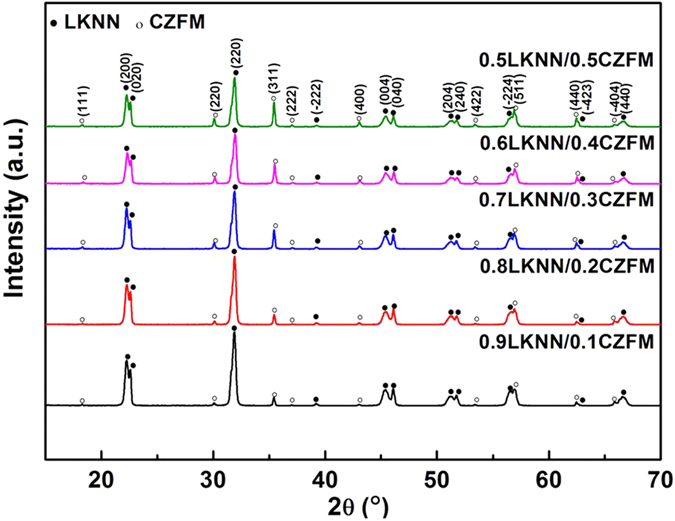
X-ray diffraction patterns of the LKNN/CZFM laminated composites.

**Figure 2:**
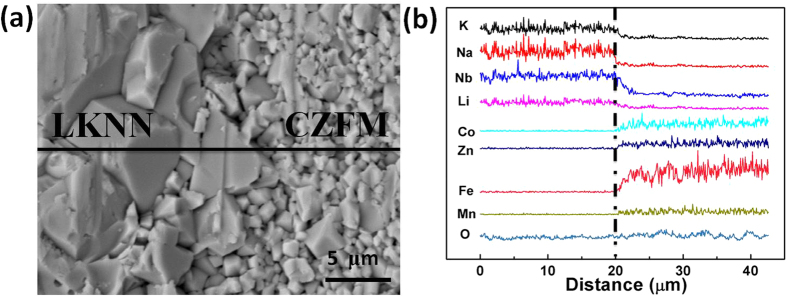
(**a**) SEM analysis result of the representative 0.9LKNN/0.1CZFM laminated composite; (**b**) Element distribution across the boundary, where the upright dot line indicates the average boundary.

Figure 1 shows the X-ray diffraction patterns of the sintered LKNN/CZFM composites. It is clear that only the expected phases of LKNN and CZFM can be detected in all the samples and the intensity of spinel peaks increases and the intensity of LKNN peaks decreases with increasing the mass ratio of CZFM. And both phases of LKNN and CZFM are in perovskite structure and spinel structure without any trace of secondary phase within the resolution of XRD instrument, indicating that there is no chemical reaction occurring between the LKNN phase and CZFM phase. The SEM image of the cross section for the representative 0.9LKNN/0.1CZFM laminate composite is shown in Fig. 2(a), demonstrating a relatively dense microstructure of the two individual phases with 10–15 μm LKNN grains and 2–5 μm CZFM grains. Figure 2(b) shows the EDS analysis of the above sample at the interface of the two phases. It can be found that a little interdiffusion occurs at the interface of the two phases. The element atomic ratios at the two sides of the boundary are consistent with the formula LKNN and CZFM, which is consistent with the above XRD analysis. And the two phases of LKNN and CZFM can coexist in the laminated structure. The SEM images of the cross section for the representative multilayer 0.9LKNN/0.01CZFM composite made by LTCC technology are shown in Fig. S1. It can be found that the multilayer structure of the composite is very clear and dense, which confirms that the as-prepared LKNN/CZFM composites are suitable for LTCC technology.

**Figure 3:**
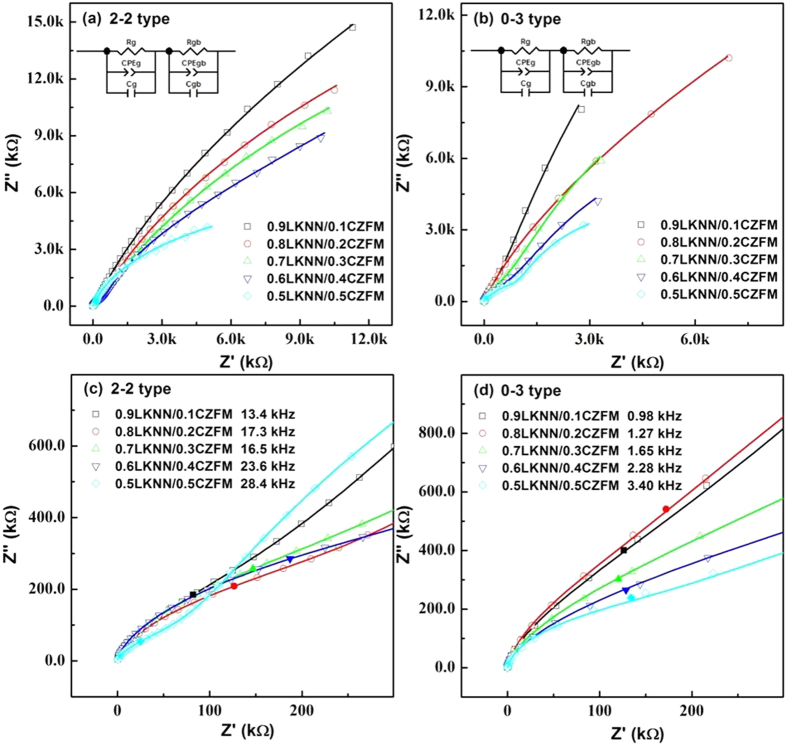
Complex impedance plots of LKNN/CZFM ceramics, and the inset exhibits the fitting equivalent circuit, where the hollow symbols are the experimental data and the lines are fitting curves: (**a**) 2-2 type; (**b**) 0-3 type. The enlarged view of the high frequency curves, where the solid symbols corresponding the characteristic frequency of grain: (**c**) 2-2 type; (**d**) 0-3 type.

Figure 3 shows the complex impedance plots for the LKNN/CZFM composites with different mass ratios of CZFM. The complex impedance plots are characterized by successive semi-circular arcs of grain (high frequencies), grain boundary (low frequency). All the experimental data were fitted based on the equivalent circuit, which was composed of two parallel R-C-CPE units associated with grain, R_g_ C_g_ CPE_g_, grain boundary, R_gb_ C_gb_ CPE_gb_, components connected in series^28^,^29^,^30^. The C_g_ and C_gb_ parameter are identified as the capacitance of grain and grain boundary, respectively^31^. The constant phase element (CPE) is used due to the non-ideal behavior of the capacitance^32^. The equivalent circuit and the fitting results are also shown in Fig. 3(a) and (b), and the excellent agreement of the fitted curves with the experimental data suggestes the validation of the equivalent circuit. The characteristic frequencies of grain and grain boundary on complex impedance plots can be calculated with the relationship of


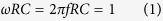


Where R is the resistance and C is the capacitance of grain and grain boundary. *ω* is the angular frequency and *f* is the characteristic frequency. The characteristic frequency of grain boundary beyond the frequency of instrument. Therefore, the characteristic frequency of grain is shown in Fig. 3(c) and (d). The characteristic frequency of grain increases with increasing the mass ratio of CZFM. The resistances of grain and grain boundary obtained from the fitted curves are listed in Table S1. It can also be found that the resistances of grain, grain boundary for the laminated composites are larger than those for the particulate composites. The introduction of laminated connectivity could enhance the resistances of the ME composites.

Figure S2 shows the dielectric constant (*ɛ*′) and dielectric loss (*tanδ*) of the KNN/CZFM composites which were measured as a function of frequency, in the range of 20 Hz and 1 MHz. It can be seen that all the samples show similar dielectric behavior and the values of *ɛ*′ and *tanδ* decrease gradually with the evolution of frequency. The *ɛ*′ of the LKNN/CZFM composites become weaker and weaker while *tanδ* gets stronger with increasing the mass ratio of CZFM at lower frequencies. They decrease with increasing the frequency and finally become almost a constant in the higher frequency range (>100 kHz). At lower frequencies, high values of *ɛ*′ are due to the presence of the different types of polarization (ionic, electronic, interfacial, and dipolar) in the materials^33^. The different resistivities of LKNN and CZFM cause localized accumulation of charges under the influence of electric field, which results in the interfacial polarization (also named as Maxwell-Wagner polarization)^34^ and seriously influences the low-frequency *ɛ*′. Thus, the interfacial polarization is the main polarization of the LKNN/CZFM laminated composites. With increasing the frequency, some of the above mentioned polarizations are unable to be in step with the frequency of the applied electric field and may have less contribution to *ɛ*′. The *tanδ* is inversely proportional to frequency (*tanδ* ≅  *γ/ωε*_*0*_*ε*_*s*_), which explains why the *tanδ* decreases with frequency at lower frequencies^35^. It can be obviously seen that the laminated composites possess the smaller values of *tanδ* compared with the particulate composites, which is due to the large resistivities of the laminated composites.

**Figure 4:**
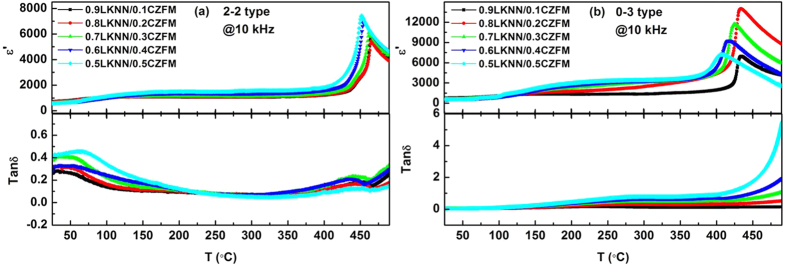
Temperature dependence of the dielectric constant (*ɛ*′) and dielectric loss (*tanδ*) of the LKNN/CZFM composites with different mass ratios of CZFM: (**a**) 2-2 type; (**b**) 0-3 type.

Figure 4 presents the temperature dependence of the dielectric constant (*ɛ*′) and dielectric loss (*tanδ*) up to 10 kHz frequency of the LKNN/CZFM composites in a temperature range from 25 °C to 480 °C with different mass ratios CZFM. Similar to the pure KNN, the LKNN/CZFM composites show two dielectric peaks, which correspond to the polymorphic phase transition (PPT) temperature and the Curie temperature (*T*_*C*_), respectively^34^. For the LKNN/CZFM composites, the two transitions occur at approximately 80 °C and 463 °C. In the *ɛ*′ curves, the peaks of LKNN/CZFM composites become broader gradually and shift to the low temperature with increasing the mass ratio of CZFM. The results is due to the ferroelectricity of LKNN is diluted by the ferromagnetic phase. The *tanδ* was also affected noticeably by the addition of CZFM. The *tanδ-T* curves of all the laminated composites exhibit two strong dielectric loss peaks at the Curie temperature (*T*_*C*_) and the orthorhombic-tetragonal (PPT) temperature, respectively. Moreover, all the laminated composites show a relatively low dielectric loss compared with the particulate composites through a broad temperature range. For the particulate composites, the *tanδ* increases gradually with the mass ratio of CZFM, and larger than that of the laminated composites. High Curie temperature and low dielectric loss enhance the temperature stability of electrical properties.

**Figure 5:**
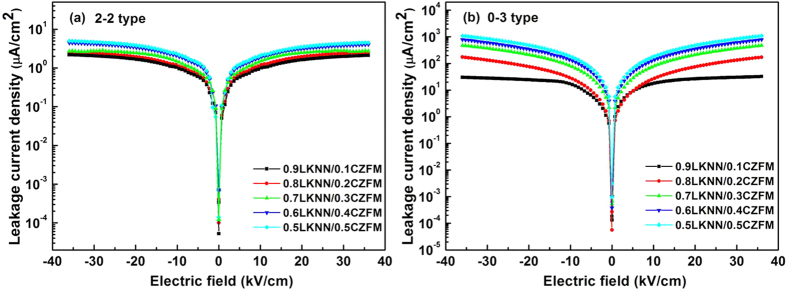
Leakage current density vs electric field for the LKNN/CZFM composites with different mass ratios of CZFM: (**a**) 2-2 type; (**b**) 0-3 type.

**Figure 6:**
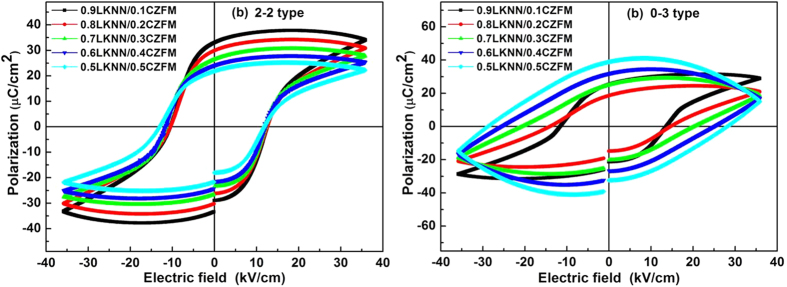
Polarization hysteresis (P-E) loops of the LKNN/CZFM composites with different mass ratios of CZFM: (**a**) 2-2 type; (**b**) 0-3 type.

Figure 5 presents the leakage current density vs Electric field (*J-E*) curves for LKNN/CZFM composites with different mass ratios of CZFM. It can be clearly found that the leakage current density of the LKNN/CZFM composites enhances with increasing the mass ratio of CZFM, and the laminated composites have lower leakage current values compared with the particulate composites. Figure 6 reveals the polarization hysteresis (*P-E*) loops of the LKNN/CZFM composites. Compared with the particulate composites, the well-saturated *P-E* loops of the laminated composites indicate an excellent ferroelectric behavior due to their lower leakage current density. With increasing the mass ratio of CZFM, the saturation polarization (*P*_*s*_) of the composites decreases while the coercive field (*E*_*c*_) increases, implying that the ferroelectricity of the composites is diminished. The results are consistent with the above leakage current analysis results.

Figure S3 shows the magnetic hysteresis loops of the LKNN/CZFM laminated composites. Notably, all the composites show obviously enhanced ferromagnetic properties and the magnetic properties are strongly dependent on the mass ratio of CZFM. As expected, the soft ferromagnetic property and higher saturation magnetization (*M*_*s*_) values can be obtained with increasing the mass ratio of CZFM, because these parameters depends on the total amount of ferromagnetic phase.

Measuring the characteristic parameters of piezoelectric materials is very important, particularly the piezoelectric constant (d_33_), which describes the coupling relationship between elastic properties and dielectric properties of piezoelectric material. The piezoelectric coefficient (d_33_) of the LKNN/CZFM composites is shown in Fig. S4. A maximum piezoelectric constant of 186 pC/N is obtained for the 0.9LKNN/0.1CZFM laminated composites. It can be seen that the laminated composites have larger *d*_*33*_ values than the particulate composites because the laminated connectivity can increase the resistivity, which makes the electrical poling sufficient and thus enhances the *d*_*33*_ values. High piezoelectric coefficient is contribution to large strain. Figure S5(a) and (b) displays the field-induced strain curves of the LKNN/CZFM composites measured at room temperature. Compared with the particulate composites, the laminated composites possess large typical strain curves with a butterfly shape because of their higher resistivities and lower leakage currents. The strain decreases significantly with increasing the mass ratio of CZFM, which is due to the fact that the addition of CZFM disrupts the ferroelectricity of the composites. The *S*_*max*_/*E*_*max*_ values of the LKNN/CZFM composites as a function of the mass ratio of CZFM are also depicted in the illustration (Fig. S5(c) and (d)). A large *S*_*max*_/*E*_*max*_ value of 518 pm/V can be obtained for the 0.9LKNN/0.1CZFM laminated composites at an applied electric field of 36 kV/cm. For better comparison, the LKNN/CZFM composites with other reported single-phase ceramics are listed in Table S2^36^,^37^,^38^,^39^,^40^. To our knowledge, the large strain of the LKNN/CZFM composites for this work are large compared with those of the reported ceramics, which implies that the as-prepared LKNN/CZFM composites can be advantageous for industrial applications.

**Figure 7:**
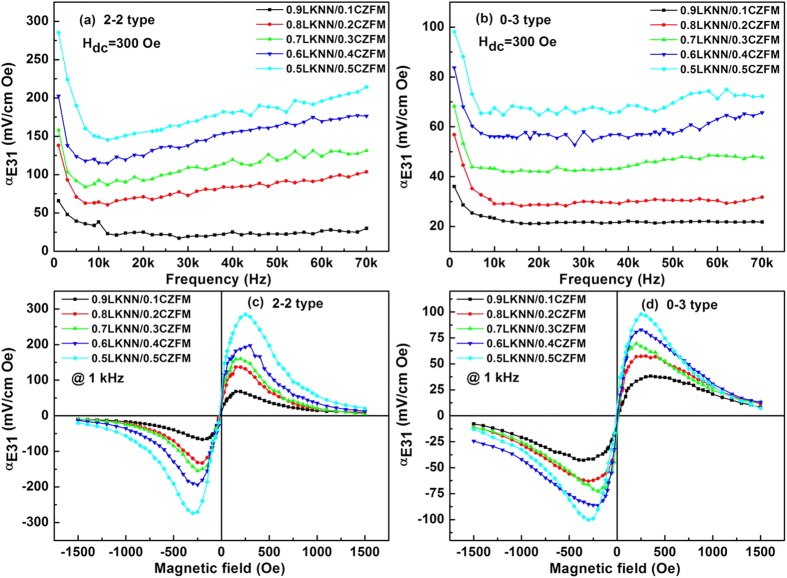
Transverse ME coefficient (*α*_*E31*_) of the LKNN/CZFM composites as functions of frequency (**a**,**b**) at 300 Oe, and magnetic field (**c**,**d**) at 1 kHz.

Figure 7(a) and (b) show the transverse ME coefficient (*α*_*E31*_) at the frequencies ranging from 1 to 70 kHz at a DC magnetic field of 300 Oe for the LKNN/CZFM composites. The direct ME effect mainly refers to the induced electric polarization under a magnetic field^41^. The direct ME effect in the composites is associated with the domain movement in the ferromagnetic phase. At lower frequencies, the *α*_*E31*_ of LKNN/CZFM composites become stronger with increasing the mass ratio of CZFM. As increasing the frequency, the *α*_*E31*_ of the laminated composites decrease gradually and then tend to stable, which exhibit a maximum value at 1 kHz. The maximum transverse ME coefficient of *α*_*E31*_ is 279 mV/cm Oe, which is obtained for the 0.5LKNN/0.5CZFM laminated composite at 1 kHz, which is approximately three times as large as that of the particulate composites (*α*_*E31*_ = 98 mV/cm Oe).

Figure 7(c) and (d) shows the *DC* magnetic field dependence of the transverse ME coefficient (*α*_*E31*_) of the laminated composites and the particulate composites at 1 kHz. The maximum ME coefficient of the laminated composites reaches up to 285 mV/cm Oe at 300 Oe, which is more than three times as large as that of the particulate composites (*α*_*E31*_ = 93 mV/cm Oe). This huge discrepancy indicates a better coupling between the LKNN layer and CZFM layer in the laminated composites compared with the coupling in the particulate composites.

**Figure 8:**
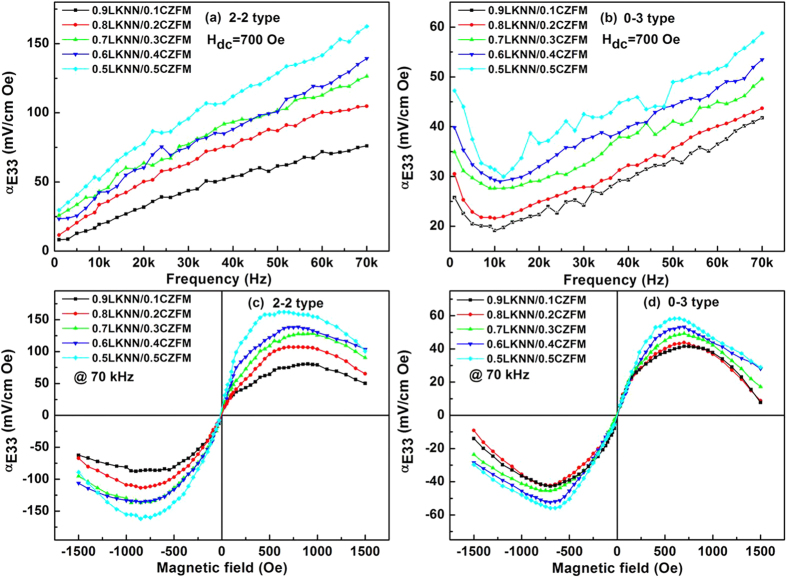
Longitudinal ME coefficient (*α*_*E33*_) of the LKNN/CZFM composites as functions of frequency (**a**, **b**) at 700 Oe, and magnetic field (**c**, **d**) at 70 kHz.

Figure 8(a) and (b) show the dependence of longitudinal ME coefficient (*α*_*E33*_) at the frequencies ranging from 1 to 70 kHz at a DC magnetic field of 700 Oe for the LKNN/CZFM composites. The ME coefficient enhances significantly with the increasing the mass ratio of CZFM. In addition, the ME coefficient also increases with the frequency for the laminated composites. Meanwhile, the ME coefficient of the particulate composites decrease firstly and then increase gradually. The largest longitudinal ME coefficient of *α*_*E33*_ = 163 mV/cm Oe is obtained for the 0.5LKNN/0.5CZFM laminated composites, which is more than three times as large as that of the particulate composites (*α*_*E33*_ = 58 mV/cm Oe).

Figure 8(c) and (d) shows the DC magnetic field dependence of the longitudinal ME coefficient (*α*_*E33*_) for the LKNN/CZFM composites at 70 kHz. The dependence of the ME response indicates that the composites on the magnetic bias field is dominated by the field-dependent magnetostriction of the CZFM layer. The *α*_*E33*_ of the LKNN/CZFM laminated composites enhances with increasing the magnetic field and the mass ratio of CZFM. In higher magnetic field range, the magnetostriction approaches to its saturated state, and *α*_*E33*_ is expected to slightly vary with magnetic field.

The difference between *α*_*E31*_ and *α*_*E33*_ dependence of magnetic field is due to the different magnetostrictions arising from the out-of-plane bias and in-plane bias in such anisotropic laminated composite samples^42^,^43^. Moreover, for the laminated composites, the disk-like samples lead to a larger area under the magnetic field for measuring *α*_*E33*_ than measuring *α*_*E31*_. The applied *H* field has not the same effect on the direct ME coefficient if applied along Direction 3 or 1, which is due to quite different demagnetizing factors acting along both directions. However, the field-dependent direct ME coefficient for the particulate composites exhibit some different behaviors. The angle variation not only affects piezoelectric properties but changes the magnetostriction, resulting in the ME anisotropy in the laminated composites, which is quite different from the much higher isotropic character of the particulate composites.

In the ME composites, the ME effect is generated as a product effect of the magnetostriction of magnetic phase and the piezoelectricity of ferroelectric phase, which is strongly dependent on the relative mass ratio of LKNN and CZFM. Both *α*_*E31*_ and *α*_*E33*_ of the composites nearly linearly increase with increasing the mass ratio of CZFM and reach the maximum value for the 0.5LKNN/0.5CZFM composite at 1 kHz. This phenomenon is attributed to the increased effective magnetostriction of the LKNN/CZFM composites due to the increased mass ratio of CZFM. However, the decreased mass ratio of LKNN limits the piezoelectric properties of the composites. This characteristic similar with that of the PZT-PVDF/Terfenol-D composites^14^ and the PZT-PVDF/PVDF-Terfenol-D composites^15^.

It is known that leakage current would also contribute to polarization^44^,^45^. Consequently, the ME properties of ME materials might occur from leakage current in the case of the change of resistance with the variation of external magnetic field^46^. To confirm the contribution of leakage current on the ME properties of the LKNN/CZFM composites, the magneto-resistances of the LKNN/CZFM composites were measured by varying the bias magnetic field (from −1500 Oe to 1500 Oe) at a fixed bias voltage of 100 V, as shown in Fig. S6. Almost no changes can be found for the resistances of the LKNN/CZFM composites with the variation of the external magnetic field, which confirms that there is no extra contribution of leakage current on the ME properties of the LKNN/CZFM composites. The as-measured ME coefficients are the intrinsic ME coupling of the LKNN/CZFM composites.

In order to further investigate the ME coefficient of LKNN/CZFM laminated composites, the theoretical estimation of the ME coefficient (*α*_*E31*_) is given. For bulk ceramic laminated composites, the transverse ME coefficient can be expressed as^47^:


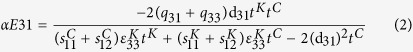


Where *t*^*K*^ and *t*^*C*^ are the volume fractions of LKNN and CZFM, i.e., the thickness fractions of the two phases. 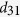 and 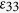 are piezoelectric and dielectric constants of LKNN. 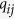 is *dλ/dH* of CZFM in different directions. 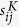 and 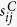 are compliances of LKNN and CZFM. Using the sample parameters for LKNN and CZFM (listed in Table S3^48^,^49^), the theoretical *α*_*E31*_ = 643 mV/cm Oe is obtained. However, our experimental result is only about 285 mV/cm Oe, which is approximately 45% of the estimated theoretical value. This phenomenon is similar with the bulk composite of PZT film on CFO ceramic^47^. However, this value is larger than that of the bulk composite of PZT film on CFO ceramic, which is less than 4% of the estimated theoretical value. The theoretical estimation is made by assuming a perfect interface coupling between the LKNN phase and CZFM phase. Actually, the interface coupling is not perfect due to some inevitable defects at the interfaces. For better comparison, the largest ME coefficient of the LKNN/CZFM composites and some reported bulk ceramic ME composites are listed in the Table S4^50^,^51^,^52^,^53^,^54^,^55^. It can be found that the largest ME coefficient of the laminated LKNN/CZFM composites for this work is larger than those of the reported lead-free bulk ceramic ME composites and comparable to those of PZT-based ME composites. To the best of our knowledge, the largest ME coefficient of the as-prepared laminated LKNN/CZFM composites is the highest for the lead-free bulk ceramic ME composite so far.

## Conclusions

Multiferroic laminated LKNN/CZFM composites has been successfully prepared by the conventional solid-state sintering method. The LKNN and CZFM phases can coexist in the composites. The electrical and ME properties of the laminated LKNN/CZFM composites are more excellent than those of the particulate composites. Compared with the particulate composites, the laminated composites display larger piezoelectric coefficients (d_33_), larger ME coefficients and lower leakage currents. It can be seen that the piezoelectric coefficient (d_33_) and the *S*_*max*_/*E*_*max*_ value decreases while the magnitude of the ME coefficient increases with increasing the mass ratio of CZFM. A large S_max_/E_max_ value of 518 pm/V can be obtained for 0.9LKNN/0.1CZFM at an applied electric field of 36 kV/cm. For the laminated 0.5LKNN/0.5CZFM composite, the transverse ME coefficient (*α*_*E31*_) reaches up to 285 mV/cm Oe, which is the highest value for the lead-free bulk ceramic ME composites so far.

## Methods

Lead-free ME laminated composites of Li_0.058_(Na_0.535_K_0.48_)_0.942_NbO_3_/Co_0.6_Zn_0.4_Fe_1.7_Mn_0.3_O_4_ were prepared by the conventional solid-state method. The starting materials of K_2_CO_3_ (99.0%), Na_2_CO_3_ (99.8%), Li_2_CO_3_ (98.0%) and Nb_2_O_5_ (99.5%) were dried to remove the absorbed moisture, weighed according to the stoichiometric ratio of LKNN, ball-mixed in alcohol for 24 h. The mixed powders were dried and then calcined at 850 °C for 4 h to obtain LKNN powder. Meanwhile, Co_3_O_4_ (99.0%), ZnO (99.0%), MnO_2_ (98.0%) and Fe_2_O_3_ (99.0%) were weighed according to the stoichiometric ratio of CZFM, ball-mixed thoroughly in alcohol and calcined at 1100 °C for 8 h to obtain CZFM powder. In order to co-fire the two phases together, the sintering temperature of the two phases should be consistent. Thus, 0.3 wt% Li_2_CO_3_ was added into CZFM powder as sintering aids to lower its sintering temperature. The LKNN powder and CZFM powder were ball-milled and granulated along with gradually adding the same amount of PVA aqueous solution, respectively. According to different mass ratios, the granulated LKNN powder and CZFM powder were weighed and poured into the die in the order of LKNN, CZFM and LKNN, and then the laminated composite powders were pressed together. The heavily pressed through the cold isostatic pressing method was used in order to ensure the same thickness of the top and bottom LKNN layers. According to a series of sintering experimental results, as shown in Fig. S7, the optimum sintering temperature of the laminated LKNN/CZFM composites is 1030 °C for 4 h. For comparison, the particulate composites of LKNN/CZFM were also sintered at the same condition.

The phase compositions of the LKNN/CZFM composites were determined using an X-ray diffractometer (D/max 2200 pc, Rigaku, Tokyo, Japan). The microstructure of the LKNN/CZFM composites was observed by a scanning electron microscope (JSM-6390A, JEOL Ltd., Tokyo). The dielectric properties of the composites were measured from 20 Hz to 1 MHz using an impedance analyzer (E4990A, Agilent, Palo Alto, CA). The temperature dependence of dielectric properties was measured with an LCR meter (3532-50, Hioki, Ueda, Japan). The polarization hysteresis loops, leakage current densities and strain curves of the LKNN/CZFM composites were characterized using a ferroelectric test system (Premier II, Radiant, USA). The magnetic hysteresis loops of the LKNN/CZFM composites were measured by a vibrating sample magnetometer (Lake Shore 7410, Westerville, OH). The piezoelectric coefficients (d_33_) of the LKNN/CZFM composites were measured by a quasi-state *d*_*33*_ meter (ZJ-3A, Institute of Acoustics, Chinese Academy of Science) after polarization procedure. The magneto-resistance of the LKNN/CZFM composites were measured by varying the bias magnetic field (from −1500 Oe to 1500 Oe) by a high resistance meter (Keithley 6517B, Ohio, USA). ME measurements were done by varying the bias magnetic field (from −1500 Oe to 1500 Oe) under a superimposed ac magnetic field of 1 Oe, in the frequency range of 1 kHz–70 kHz, generated by Helmholtz coils. The charges generated from the samples were collected by a charge amplifier (DSC3062, Beijing, China). The schematic illustration of the testing setup for ME measurement is shown in Fig. S8. When the polarization direction (Direction 3) was parallel to the magnetic field, i.e., *θ* = 0°, the longitudinal ME sensitivity *α*_*E33*_ = *δE*_*3*_*/δH*_*3*_ was obtained. When the polarization direction was perpendicular to the magnetic field, i.e., *θ* = 90°, the transverse ME coefficient *α*_*E31*_ = *δE*_*3*_*/δH*_*1*_ was measured. For the detailed description, a coordinate system is assumed with the sample in the (1, 2) plane. The sample is poled with an electric field E along the Direction 3. ME coupling is estimated from the induced field *δE* across the sample that is subjected to an ac magnetic field *δH* in the presence of a bias field H. Basic relations for ME coefficients are obtained for two orientations of *H* and *δH*: along Direction 3 (longitudinal to *δE*) or along Direction 1 (transverse to *δE*), as depicted in Fig. S9.

## Additional Information

**How to cite this article**: Yang, H. *et al*. High Curie temperature and enhanced magnetoelectric properties of the laminated Li_0.058_(Na_0.535_K_0.48_)_0.942_NbO_3_/Co_0.6_Zn_0.4_Fe_1.7_Mn_0.3_O_4_ composites. *Sci. Rep.* **7**, 44855; doi: 10.1038/srep44855 (2017).

**Publisher's note:** Springer Nature remains neutral with regard to jurisdictional claims in published maps and institutional affiliations.

## Supplementary Material

Supplementary Information
