# Supplementary material for: High Curie temperature and enhanced magnetoelectric properties of the laminated Li0.058(Na0.535K0.48)0.942NbO3/Co0.6 Zn0.4Fe1.7Mn0.3O4 composites
Source: Sci Rep. 2017 Mar 24;7:44855. doi: 10.1038/srep44855 (PMC5364461; doi:10.1038/srep44855)
Supplement: Supplementary Information [file srep44855-s1.doc]

**On-line Supplementary information for**

High Curie temperature and enhanced magnetoelectric properties of the laminated Li_0.058_(Na_0.535_K_0.48_)_0.942_NbO_3_/Co_0.6_Zn_0.4_Fe_1.7_Mn_0.3_O_4_ composites

Haibo Yang[[1]](#footnote-2)*, Jintao Zhang, Ying Lin, Tong Wang

*School of Materials Science and Engineering, Shaanxi University of Science and Technology, 710021，Xi’an, PR China.

AUTHOR INFORMATION

Corresponding Author

(*Haibo Yang) E-mail: yanghaibo@sust.edu.cn.

Table S1 Resistances (grain resistances R_g_, grain boundary resistances R_gb_) of the LKNN/CZFM composites.

| **CZFM**  **Content (wt%)** | **R_g_ (Ω) (2-2 type)** | **R_gb_** **(Ω) (2-2 type)** | **R_g_ (Ω) (0-3 type)** | **R_gb_** **(Ω) (0-3 type)** |
| --- | --- | --- | --- | --- |
| 0.1 | 2.49×10^5^ | 1.28×10^8^ | 1.91×10^5^ | 9.08×10^7^ |
| 0.2 | 4.12×10^5^ | 7.72×10^7^ | 2.21×10^5^ | 7.54×10^7^ |
| 0.3 | 4.17×10^5^ | 6.32×10^7^ | 3.32×10^5^ | 5.41×10^7^ |
| 0.4 | 6.45×10^5^ | 5.02×10^7^ | 4.27×10^5^ | 4.59×10^7^ |
| 0.5 | 5.52×10^5^ | 3.01×10^7^ | 3.60×10^5^ | 2.04×10^7^ |

Table S2 Comparison of the LKNN/CZFM laminated composites with other reported ceramics.

| **Material** | **S_max_/E_max_ (pm/V)** | **Reference** |
| --- | --- | --- |
| [KNLi(Nb,Ta,Sb)]O_3_ | 750 | [36] |
| NKNS-LT-BZ | 508 | [37] |
| KNN-LiSbO_3_ | 330 | [38] |
| KNN-LiTaO_3_ | 310-400 | [39] |
| PZT-SKN | 779 | [40] |
| LKNN/CZFM | 518 | This work |

Table S3 Material parameters (compliance coefficient s_ij_, piezomagnetic coupling q_ij_, piezoelectric coefficient d_31_ and petmittivity ε_33_) for potassium sodium niobate (LKNN), cobalt ferrite (CFO).

| **Material** | **S_11_**  **(10^-12^m^2^/N)** | **S_12_**  **(10^-12^m^2^/N)** | **q_31_**  **(10^-12^m/A)** | **q_33_**  **(10^-12^m/A)** | **d_31_**  **(10^-12^m/V)** | **ε_33_** | **Reference** |
| --- | --- | --- | --- | --- | --- | --- | --- |
| **LKNN** | 11.4 | -4.21 | - | - | -59 | 480 | [48] |
| **CFO** | 6.5 | -2.4 | 556 | -1880 | - | - | [49] |

Table S4 Some reported bulk ceramic ME composites and their largest ME coefficients

| **Laminated composite** | | |  | **Particulate composite** | | |
| --- | --- | --- | --- | --- | --- | --- |
| **Composite** | **ME coefficient (mV/cm Oe)** | **Reference** |  | **Composite** | **ME coefficient (mV/cm Oe)** | **Reference** |
| **LKNNTS/NCZF** | 133 | [22] |  | **BNT-BT/CFO** | 10.08 | [50] |
| **PZT/CFO** | 163 | [51] |  | **KNN-LS/NZF** | 20.14 | [52] |
| **PFT/CFO** | 200 | [53] |  | **CFO/P(VDF-TrFE)** | 40 | [54] |
| **LKNNS-NZF/Ni/**  **LKNNS-NZF** | 261.3 | [52] |  | **CFO/PZT** | 92 | [55] |
| **LKNN/CZFM** | 285 | This work |  | **LKNN/CZFM** | 98 | This work |


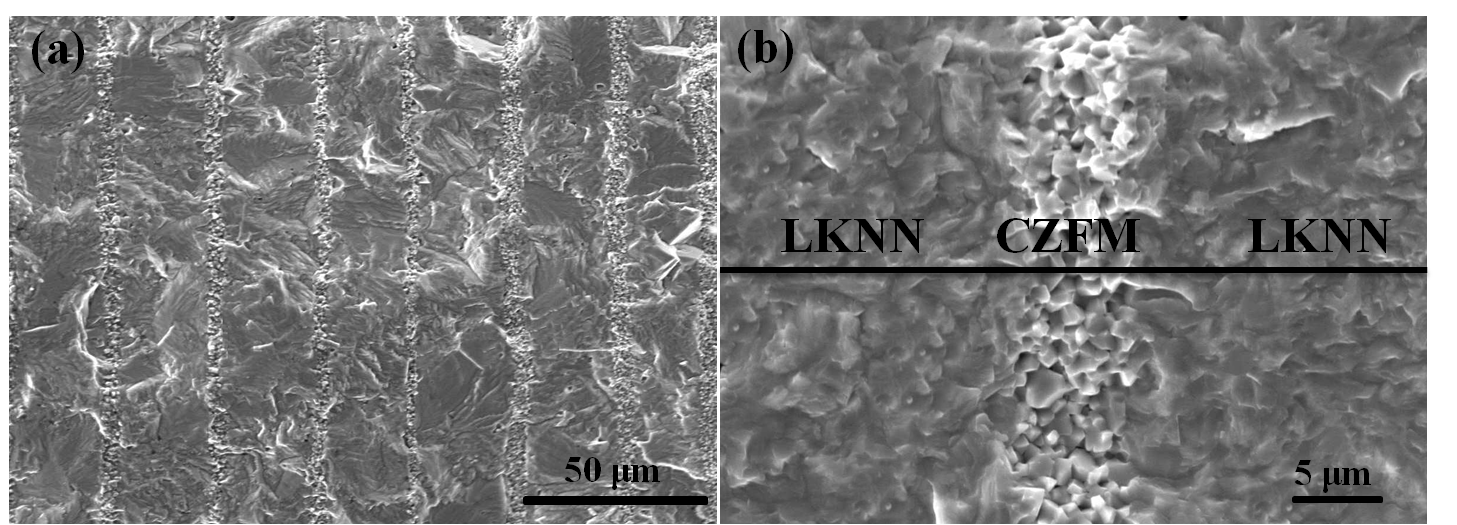


Fig. S1 SEM images of the cross section for the representative multilayer 0.9LKNN/0.1CZFM composite made by LTCC technology.


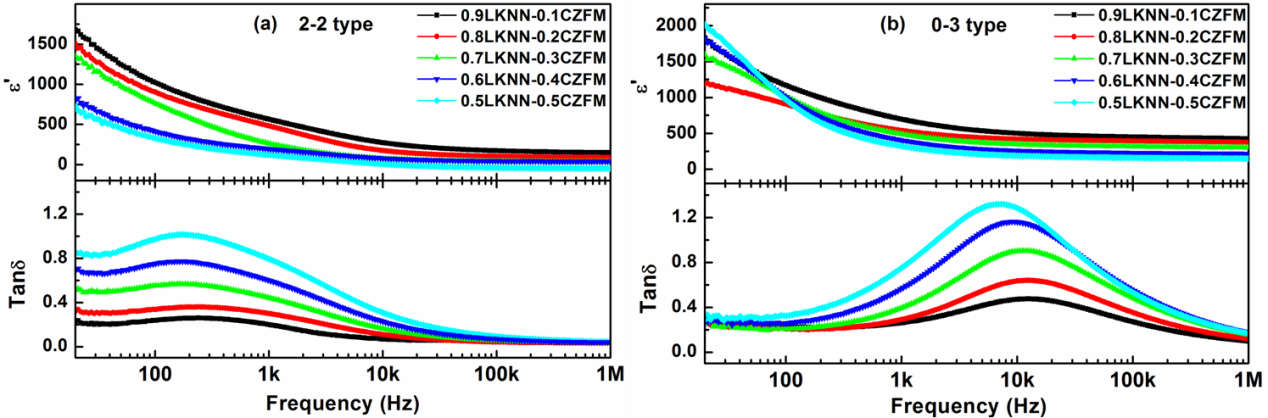


Fig. S2 Frequency dependence of the dielectric constant (*ɛ’*) and dielectric loss (*tanδ*) of the LKNN/CZFM composites with different mass ratios of CZFM: (a) 2-2 type; (b) 0-3 type.


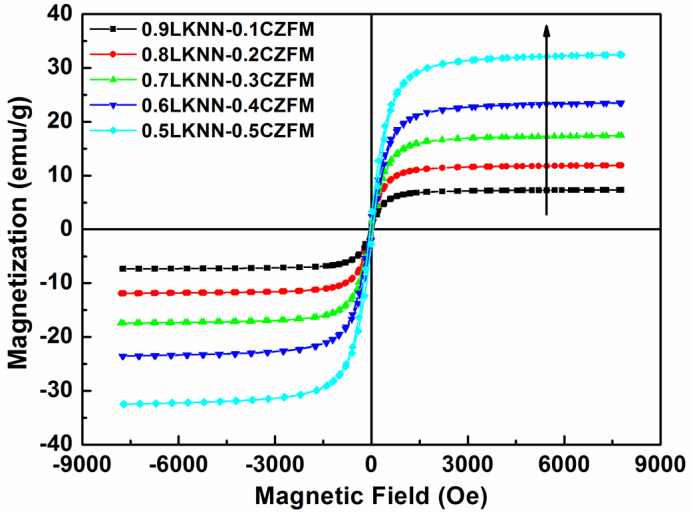


Fig. S3 Magnetic hysteresis (M-H) loops of the LKNN/CZFM laminated composites with different mass ratios of CZFM.


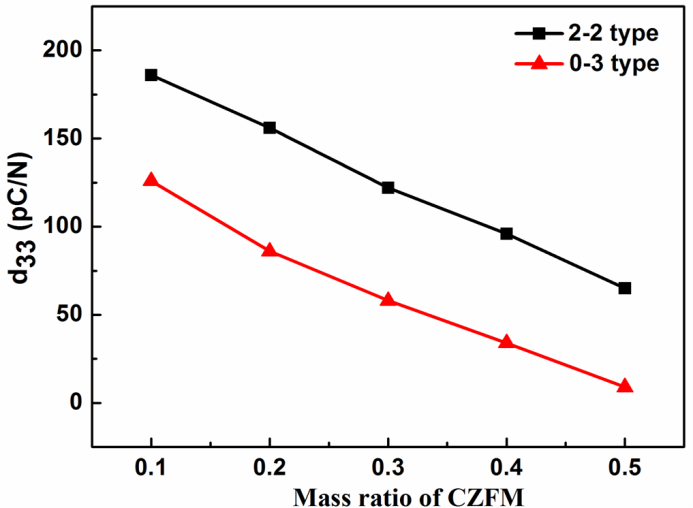


Fig. S4 Piezoelectric coefficient (*d_33_*) of the LKNN/CZFM composites.


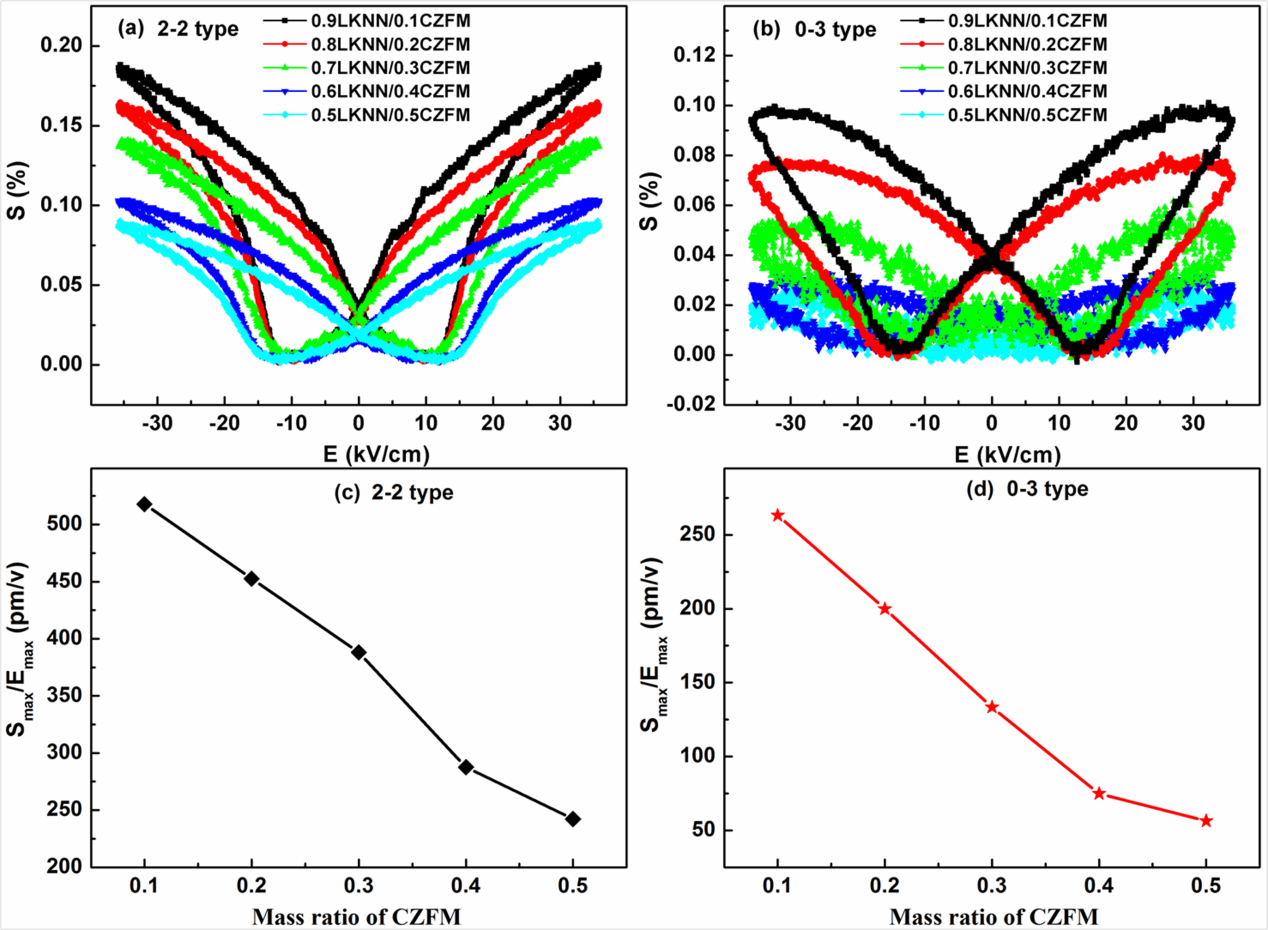


Fig. S5 Field-induced strain curves and *S_max_/E_max_* values of the LKNN/CZFM composites with different mass ratios of CZFM: (a, c) 2-2 type; (b, d) 0-3 type.


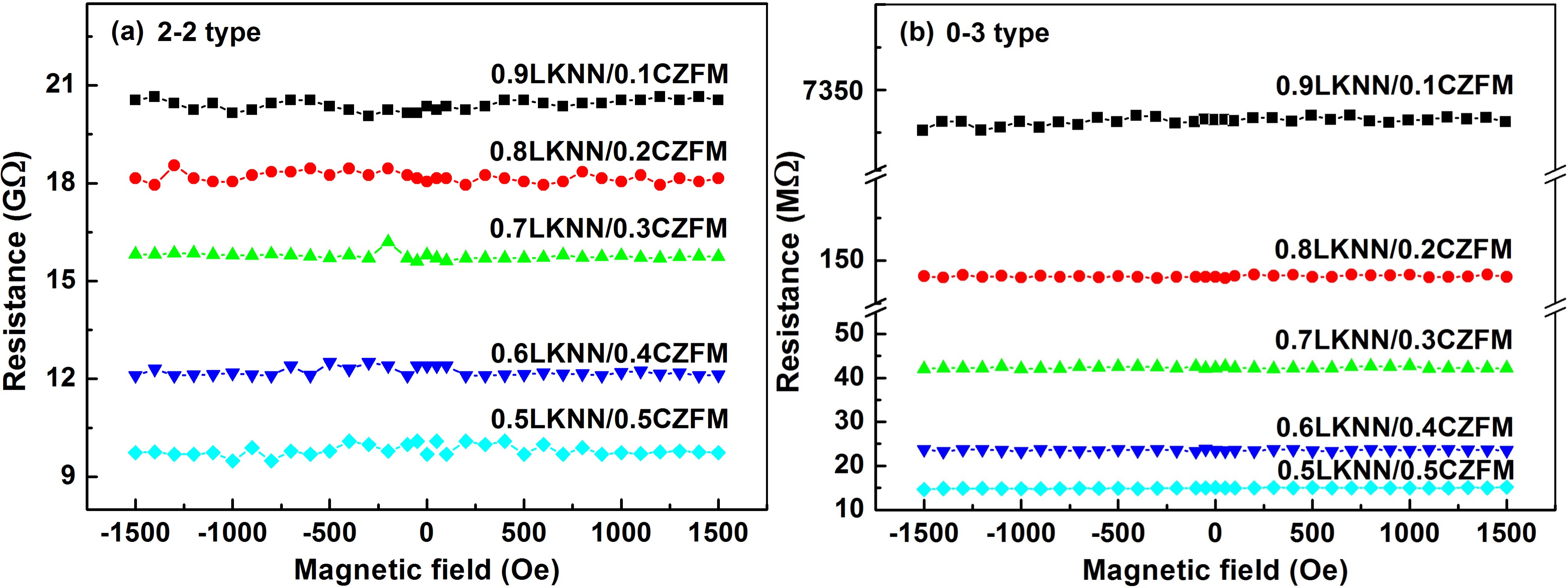


Fig. S6 Magnetic field dependence of the resistance of the LKNN/CZFM composites with different mass ratios of CZFM: (a) 2-2 type; (b) 0-3 type.


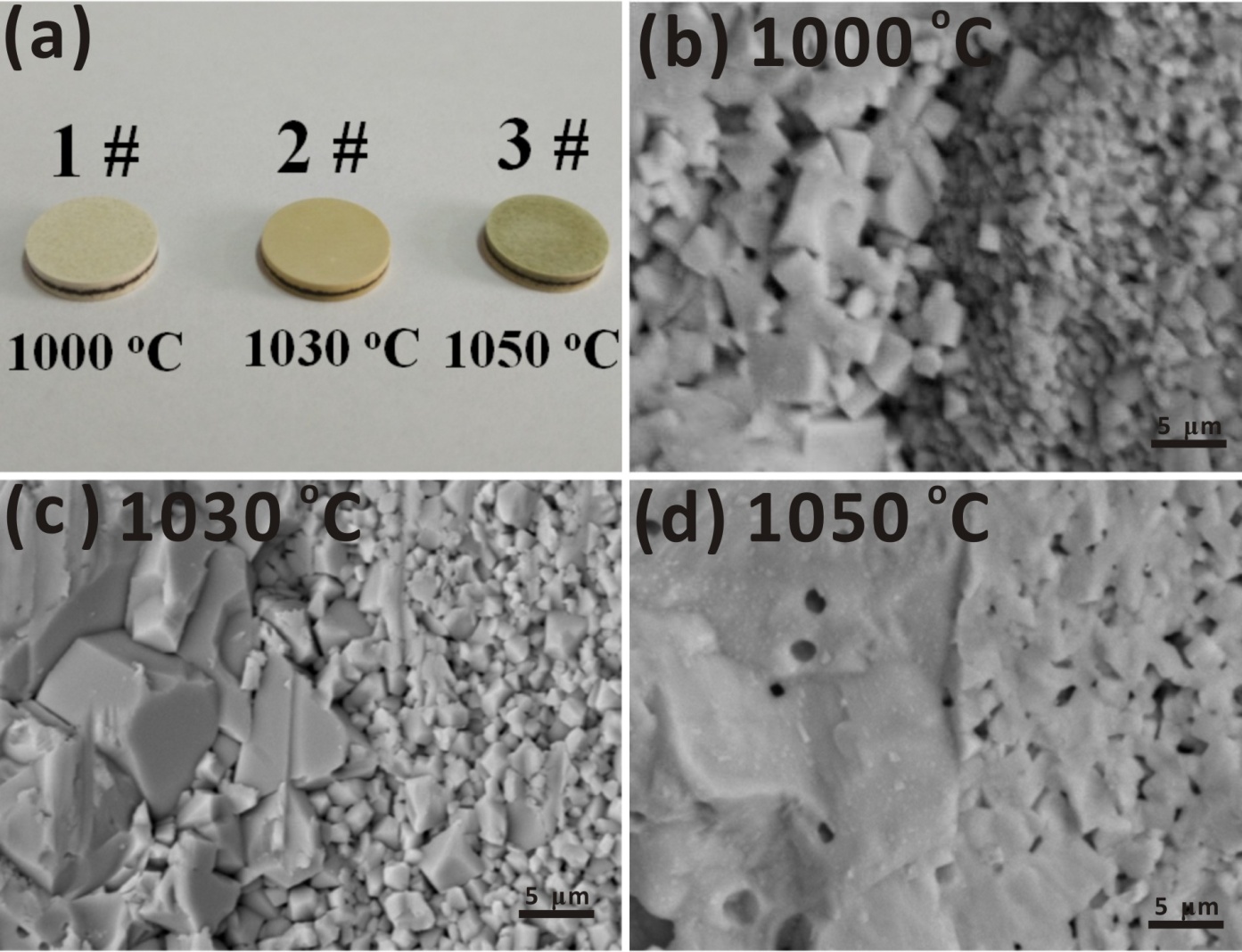


Fig. S7 Digital photo and SEM images of the representative 0.9LKNN/0.1CZFM laminated composites with the different sintering temperatures for 4 h.


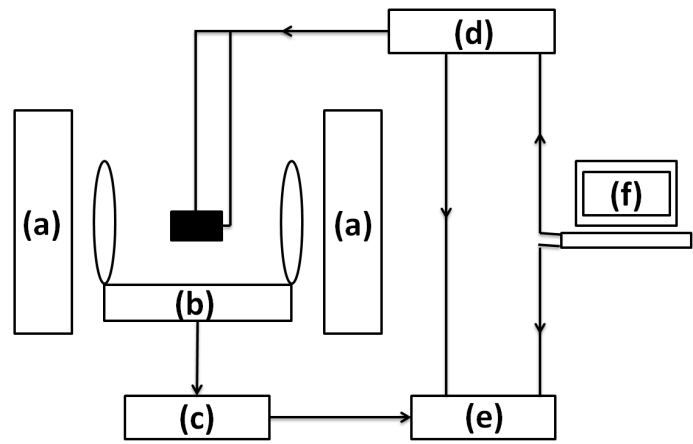


Fig. S8 Schematic illustration of the testing setup for ME measurement: (a) Electromagnet; (b) Test coil; (c) Voltage amplifier; (d) Signal generator; (e) Oscillograph; (f) Computer.


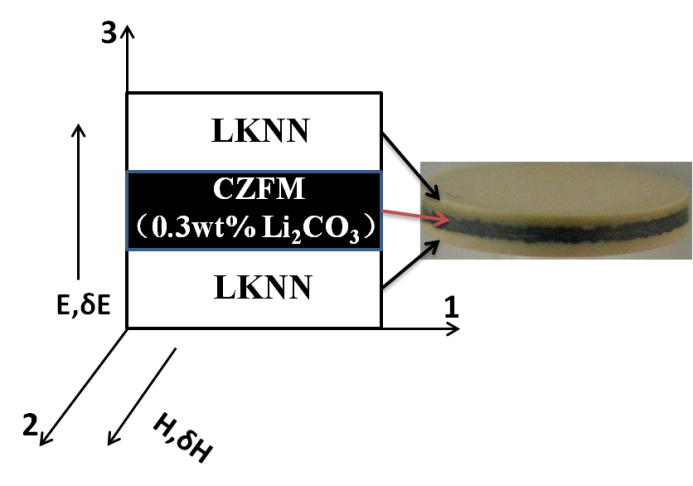


Fig. S9 Schematic structure diagram and digital photo for the LKNN/CZFM laminated composites.

1. ^^ Corresponding author. Tel: +86-29-86168688; Fax: +86-29-86168688; Email: yanghaibo@sust.edu.cn [↑](#footnote-ref-2)
